# Social and Behavioral Difficulties in 10-Year-Old Children With Congenital Heart Disease: Prevalence and Risk Factors

**DOI:** 10.3389/fped.2020.604918

**Published:** 2020-12-11

**Authors:** Isabelle Werninger, Melanie Ehrler, Flavia M. Wehrle, Markus A. Landolt, Susanne Polentarutti, Emanuela R. Valsangiacomo Buechel, Beatrice Latal

**Affiliations:** ^1^Child Development Center, University Children's Hospital Zurich, Zurich, Switzerland; ^2^Children's Research Center, University Children's Hospital Zurich, Zurich, Switzerland; ^3^Department of Neonatology and Intensive Care, University Children's Hospital Zurich, Zurich, Switzerland; ^4^Department of Psychosomatics and Psychiatry, University Children's Hospital Zurich, Zurich, Switzerland; ^5^Division of Child and Adolescence Health Psychology, Department of Psychology, University of Zurich, Zurich, Switzerland; ^6^Division of Cardiology, Pediatric Heart Center, University Children's Hospital Zurich, Zurich, Switzerland

**Keywords:** congenital heart disease, behavioral outcome, social interaction, ADHD, ASD, school-aged children

## Abstract

Children with congenital heart disease (CHD) may be at increased risk for neurodevelopmental impairments. Long-term behavioral profiles and respective risk factors are less frequently described. The aim of this study was to evaluate multidimensional behavioral outcomes and associated medical, psychological, and social risk factors in children with complex CHD. At 10-years of age, 125 children with CHD were assessed for general behavioral difficulties, attention deficit hyperactivity disorder (ADHD)-related behavior, and social interaction problems and were compared to normative data. Medical and cardiac factors, IQ, maternal mental health at 4 years of age and parental socioeconomic status were tested as predictors for all behavioral outcomes. Children with CHD showed no significant differences in general behavioral difficulties. However, increased ADHD-related symptoms (*p* < 0.05) and difficulties in social interaction (*p* < 0.05) were observed. In 23% of the children, a combination of ADHD-related symptoms and social interaction problems was reported by parents. In multivariate analyses, IQ (*p* < 0.01) and maternal mental health (*p* < 0.03) at 4 years of age were found to be predictive for all behavioral outcomes at 10 years while medical and cardiac risk factors were not. Our findings reveal significant difficulties in ADHD-related symptoms and social interaction problems with a significant comorbidity. Behavioral difficulties were not detected with a screening tool but with disorder-specific questionnaires. Furthermore, we demonstrate the importance of maternal mental health during early childhood on later behavioral outcomes of children with CHD. This underlines the importance of identifying and supporting parents with mental health issues at an early stage in order to support the family and improve the child's neurodevelopment.

## Introduction

Congenital heart disease (CHD) is the most frequent congenital malformation with an incidence of 8 per 1,000 live births (1), with approximately one third requiring cardiopulmonary bypass (CPB) surgery (2). With increased medical advances within the last decades, the majority of children with CHD who undergo open-heart surgery survive into adulthood (3). Therefore, CHD is classified as a chronic disease (4). It is thus of importance to focus on the long-term outcome of this patient population.

Studies have shown a considerable risk for cognitive, behavioral, and emotional problems in children with CHD undergoing open-heart surgery (4, 5). Neurodevelopmental impairments become increasingly apparent during school age when the child encounters new and more complex academic situations. At school age, children with CHD have a higher rate of learning difficulties, motor deficits, problems with social relationships, and poor school performance compared to healthy peers (6). A meta-analysis by Abda et al. demonstrated that ~25% of children and adolescents with CHD have significant behavioral problems (4). Interestingly, behavioral difficulties become more prominent in older children with CHD [see meta-analyses (4, 7, 8)]. The most frequent behavioral difficulties are emotional and peer problems, which can be summarized as internalizing problems (7). However, also externalizing behavioral problems, such as attention deficit hyperactivity disorder (ADHD)-related symptoms, have been described to occur more frequently in CHD children compared to controls (6, 8). Furthermore, Bellinger raised concerns about potential impairments in social cognition (9). Subsequently, some studies reported a higher risk for Autism Spectrum Disorder (ASD) and theory of mind problems as well as elevated scores in ASD screening tools (4, 10). However, little is known about behavioral comorbidities in combination with social problems and the description of social difficulties in a cohort with a broader spectrum of CHD is lacking.

To understand the underlying correlates of behavioral problems in CHD, studies have mainly focused on neonatal and perioperative risk factors (11). Among others, age at first surgery, number of surgeries, and length of intensive care unit (ICU) stay have been reported as risk factors for neurodevelopmental and behavioral impairments (6, 12, 13). However, Huisenga et al. conclude in their discussion that this association remains inconsistent across studies (6). Furthermore, IQ and socioeconomic status (SES) have been linked to child's behavior in this population as well (5, 14). There is evidence that psychological and social factors have a higher impact than the medical factors (15, 16). A striking factor impacting family well-being is parental mental health. A systematic review by Woolf-King et al. showed elevated mental health symptoms in parents, especially in mothers, of children with CHD (17). In turn, there is concern that parental mental health is associated with the children's neurodevelopmental and behavioral outcome (17). This has been shown for a cohort of infants and toddlers with CHD (18), however, insufficient information exists on an association between parental mental health and persistent behavioral problems in this population. Further, studies testing multilinear models testing all medical, psychological, and social risk factors are lacking.

We thus aimed to investigate multidimensional behavioral outcomes using a screening tool for general behavioral difficulties, and questionnaires to identify ADHD-related symptoms and social interaction problems in 10-year-old children with CHD. Further, we explored all, medical, psychological, and social risk factors, including maternal mental health, as predictors for adverse behavioral outcomes. We hypothesized that children with CHD had more difficulties across all behavioral outcomes compared to the norm and that psychological and social factors have a greater influence than medical factors on behavioral outcomes.

## Materials and Methods

### Study Population

Children with different types of CHD were recruited from the prospective cohort study *Research and Child Health Outcome* (*REACHOUT*) of the University Children's Hospital Zurich, Switzerland (19). All patients underwent CPB surgery between 2004 and 2009, at below 6 years of age. Patients with a genetic disorder or syndrome were excluded. Neurodevelopmental follow up assessments were conducted across childhood (see Supplementary Figure 1 for details). Comprehensive information on child behavior, family environment, and parental mental health were assessed by means of questionnaires at each assessment. Clinical data were prospectively collected from patient's charts.

Of 198 children eligible for the 10-year examination, 135 were assessed and behavioral outcomes were available in 125. Compared to the 73 children who were eligible for the 10-year examination but who did not attend (*n* = 63) or had no information on behavior available (*n* = 10), the 125 children had a higher IQ at 6 years of age (*p-*value = 0.001), a higher SES (*p-*value = 0.004) and a higher RACH-score (20) (*p-*value = 0.021).

### Developmental and Behavioral Assessment

The behavioral outcomes of the children with CHD at 10 years of age were assessed with the German versions of questionnaires. First, the Strength and Difficulties Questionnaire (SDQ) is a validated screening tool for general behavioral problems of children between 3 and 16 years of age (21, 22). According to Goodman, a total difficulties score, an internalizing subscale, and an externalizing subscale were calculated (23). The internalizing subscale includes the “emotional symptoms” and “peer relationship problems” subscales, and the externalizing subscale includes the “conduct problems” and “hyperactivity/inattention” subscales. Secondly, the Social Responsiveness Scale (SRS) was used to assess social interaction problems and autistic features (24). The SRS consists of a total score and five subscales: “social cognition,” “social awareness,” “social communication,” “social motivation,” and “autistic mannerism.” Thirdly, the Conners-3 questionnaire (short form) was used as a measure for ADHD-related symptoms and common comorbidities including six subscales: “inattention,” “hyperactivity/impulsivity,” “learning problems,” “executive functioning,” “aggression,” and “peer relations” (25). All 3 questionnaires were handed out to parents of children participating in the 10-year follow-up. Parents were asked to forward the SDQ and Conners-3 to the child's head teacher. In a subsample, the Conners-3 and SDQ questionnaire were further completed by the children's teachers. For all questionnaires, acceptable internal consistency has been reported (Cronbach's α > 0.7). Higher scores of the SDQ, Conners-3, or SRS indicate more behavioral problems. All three questionnaires are frequently used in both clinical practice and research [SDQ: e.g., (26, 27), SRS: e.g., (28, 29), Connors-3: e.g., (28, 30)].

As psychological predictors for the behavioral outcomes at 10 years of age, we considered maternal mental health, measured with the “Global Severity Index” of the Brief symptom inventory [BSI; Cronbach's α > 0.92; (31)], and child's intellectual abilities, measured with the German version of the Hannover-Wechsler-Intelligence Test for Preschool Age [WPPSI-III; (32)], both assessed at 4 years of age. We chose this time point because (a) pre-school age is a vulnerable time period for the child's future development (4) and (b) the response rate for the BSI was relatively low at the prior assessments. As social predictor for behavioral outcome, we included SES at CPB surgery, derived from a 6-point Likert scale of maternal education and parental occupation (range 2–12, higher scores refer to higher SES) (33).

### Statistics

For descriptive statistics, the following sample characteristics were investigated: sex, age, IQ, SES, as well as various cardiac and neonatal characteristics. Probabilities were reported for binary data, median and range for ordinal-scaled data, and mean and standard deviation for continuous data.

All behavioral outcomes were compared to normative data: To analyze general behavioral difficulties, the SDQ total difficulties score [German, age, and sex stratified normative data: (34)] and the two subscales internalizing and externalizing problems [sex stratified normative data: (23)] were categorized into normal and abnormal behavior (combined categories: slightly raised, high, very high) and were compared to the norm using two-sampled Fisher's exact tests.

To analyze ADHD-related symptoms and social impairment, *T*-scores were calculated for all Conners-3 [German and Swiss, age, and sex stratified normative data: (25)] and SRS] German, age, and sex stratified normative data: (35)] subscales and were compared to norms (*Mean* = 50, *SD* = 10) using one-sample *t*-test (for SRS) respectively Mann-Whitney *U*-tests (for Connors-3 to account for skewed data).

For teacher-reported SDQ and Conners-3, the same statistical approach was used. Since there are no norms for teachers in the reference by Janitza et al. (34), the norms by Goodman (36) were used.

To descriptively investigate behavioral comorbidities, the number of abnormal behavioral outcomes across questionnaires was reported: For SDQ and SRS, behavior was rated abnormal, if the respective total score was outside the normal range. For Conners-3, due to a missing total score in the short form, behavior was rated abnormal when 2 or more subscales scored outside the normal range. In this *post-hoc* investigation, only children with a parental rating of all 3 questionnaires were included (*n* = 88).

For the risk factor analysis, behavioral outcomes were associated with risk factors in separate multiple linear regression models. For analyses regarding SDQ and SRS, the total scores of these questionnaires were used as outcome. Due to a missing total score in the Connors-3 short form, an ordinal linear regression was calculated using the number of scales scoring outside the normal range as outcome (abnormal scale if *T* > 60; outcome ranges from 0 to 6 abnormal scales). IQ and maternal mental health (BSI Global Severity Score) at 4 years of age were included as psychological risk factors and parental SES was included as social risk factor. Cardiac and neonatal factors were chosen according to a three-step process: (1) Potential risk factors were assembled according to literature research. We considered studies with large (pooled) samples and systematic reviews (5, 6, 11, 13, 37, 38). (2) Variables with low variance and more than 20% missing data were excluded. (3) Collinearity of factors was investigated with a heterogeneous correlation matrix (see Supplementary Figure 2) including polyserial and polychoric correlations for continuous, ordinal, and binary-scaled data. In case of collinearity [*r* > 0.5, (39)], one variable was excluded according to rational considerations by our cardiologist (OK) and developmental pediatrician (BL). Concluding, the following medical and neonatal factors were considered as independent variables in our risk factor model: Gestational age (GA), birth weight (corrected for GA and sex), univentricular CHD, mean pre-operative saturation, age at first CPB, lowest perioperative temperature, and extracorporeal circulation time during the first CPB surgery, and length of hospitalization after the first CPB surgery. The BSI was missing in 24 patients, which were thus excluded from the initial risk factor analysis. However, the significance of the risk factors did not change when the sample size was increased by excluding BSI from the model.

*P-*values < 0.05 were considered statistically significant. Analyses were not corrected for multiple comparisons but have to be considered as exploratory. Statistical analyses were performed with the computing environment R (40). The STROBE guidelines for reporting observational studies were followed.

## Results

### Participant Characteristics

Participant characteristics of the 125 children with completed behavioral questionnaires are shown in Table 1. Median age of our cohort at the 10-year assessment was 10.17 years (*range* = 9.50–11.25 years). *Mean* IQ was 93.86 (*SD* = 12.99) at 4 years and 95.94 (*SD* = 13.38) at 10 years of age. Maternal mental health measured with the BSI was in the normal range with *Mean T*-score = 47.13 (*SD* = 12.21, *range* = 21–80; higher scores indicate more problems). Of the 125 children with behavioral assessment, the SDQ was completed for 123, the Conners-3 for 98 and the SRS for 105 children.

**Table 1:** Demographic and cardiac characteristics of children with CHD (*N* = 125).

**Sample characteristic**	**Univentricular (*N* = 23)**	**Biventricular(*N* = 102)**
**Innate**		
Male sex, *N* (%)	12 (52.17)	63 (61.8)
Socioeconomic status, median (range)	8 (3–12)	8 (3–12)
Caucasian race, *N* (%)	23 (100.0)	95 (93.1)
Prenatal diagnosis, *N* (%)	15 (65.2)	12 (11.8)
Gestational age, wk, mean (SD)	38.97 (1.69)	39.03 (2.27)
Birth weight, g, mean (SD)	3424.78 (736.45)	3155.94 (618.98)
Head circumference at birth, cm, mean (SD)	34.73 (1.64)	34.05 (1.70)
5-min Apgar score, median (range)	9 (8–10)	9 (1–10)
Cyanotic heart defect, *N* (%)	23 (100.0)	61 (59.8)
**Pre-operative**		
Mean pre-operative saturation, mean (SD)	83.04 (9.59)	87.47 (10.34)
Hematocrit %, mean (SD)	44.65 (4.88)	39.97 (6.57)
Pre-operative intubation, *N* (%)	2 (8.7)	18 (17.6)
RACH score, median (range)	6 (2–6)	3 (1–4)
Age at first surgery, mo, mean (SD)	2.33 (2.98)	3.81 (5.72)
**Intraoperative (first CPB surgery)**		
Lowest temperature, °C, mean (SD)	28.70 (6.26)	29.02 (3.55)
Hypothermia <27°C, *N* (%)	8 (34.8)	17 (16.7)
ECC time, min, mean (SD)	154.48 (41.30)	166.49 (76.62)
Aortic cross- clamping time, min, mean (SD)	87.29 (46.89)	95.22 (49.53)
**Post-operative**		
Clinical seizures after first surgery, *N* (%)	0 (0.0)	1 (1.0)
ECMO, *N* (%)	1 (4.5)	1 (1.0)
Cardiocirculatory resuscitation post-operative, *N* (%)	3 (13.0)	6 (5.9)
Days of intubation, mean (SD)	6.74 (4.32)	5.29 (11.46)
Length of ICU stay after first surgery, d, mean (SD)	15.61 (12.77)	11.12 (23.55)
Total length of hospitalization after first surgery, mean (SD)	54.13 (43.48)	29.03 (27.47)
Total number of CPB surgeries, median (range)	3 (2–4)	1 (1–3)
Heart insufficiency at 10-year follow- up, *N* (%)	6 (26.1)	5 (4.9)

*All pre-, intra- and post-operative variables correspond to the first CPB surgery. Data was incomplete for head circumference at birth (missings: N = 19), 5-min Apgar score (missings: N = 12), mean pre-operative saturation (missings: N = 6), and aortic cross-clamping time (missings: N = 9)*.

### Behavioral Outcomes

**Figure 1:**
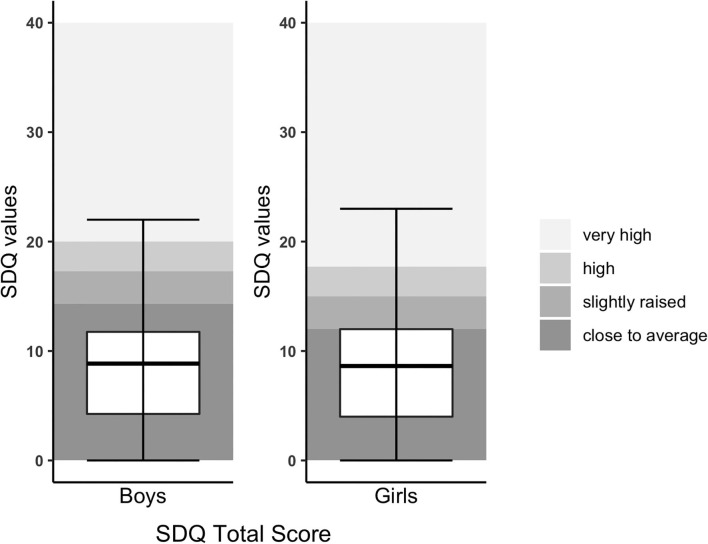
SDQ Total score of children with CHD compared to normative data. SDQ Total score was split by gender due to different cutoffs. The upper and lower borders of the box correspond to the third and first quartile. The thick line in the box represents the mean.

General behavioral difficulties measured with the SDQ revealed abnormal overall behavior in 22%, abnormal internalizing problems in 23.6%, and abnormal externalizing problems in 17.9% (Figure 1). Compared to the normative data of Janitza et al. and Goodman et al. (23, 34), differences were not significant (see Table 2). Teacher-reported SDQ revealed similar findings (see Supplementary Table 1).

**Table 2:** Parent-reported behavioral outcome of children with CHD at 10 years of age, comparison with manual norms.

	**% With abnormal score**	**Mean *T*-scores**	**Standard estimate**	***CI*-95**	***p*-values**	**Effect size**
SDQ^*^ **(*****N*** **=** **123)**						
Total Score	22.0	-	0.89	0.56–1.44	0.642	-
Internalizing Score	23.6	-	0.80	0.55–1.32	0.433	-
Externalizing Score	17.9	-	0.95	0.59–1.58	0.912	-
Conners-3^**^ **(*****N*** **=** **98)**						
Inattention	32.7	56.89	3,819	55.00–59.50	** <0.001**	0.64
Hyperactivity/impulsivity	11.2	53.94	3,802	51.00–55.00	** <0.001**	0.50
Learning problems	28.6	54.56	3,761	53.00–56.00	** <0.001**	0.48
Executive functioning	22.4	52.15	2,664	50.50–54.50	**0.013**	0.24
Aggression	22.4	55.37	1,540	58.00–61.00	** <0.001**	0.73
Peer relations	24.5	56.12	3,339	55.50–58.50	** <0.001**	0.66
SRS^**^ **(*****N*** **=** **105)**						
Total Score	33.2	52.80	2.13	50.20–55.40	**0.035**	0.21
Social cognition	24.8	55.15	4.84	53.04–57.26	** <0.001**	0.47
Social awareness	31.4	51.96	1.87	49.88–54.04	0.064	-
Social communication	26.7	53.39	2.94	51.10–55.68	**0.004**	0.29
Social motivation	25.7	53.10	2.51	50.65–55.56	**0.014**	0.24
Autistic mannerism	36.2	56.72	7.41	54.92–58.52	** <0.001**	0.72

*Scores of the SDQ are divided into normal (close to average) and abnormal (slightly raised, high, very high). Standard estimates are odds ratio for SDQ, V- values for Conners-3 and t- values for SRS. Effect sizes are reported for all significant p-values. For Conners-3 the Rosenthal r is reported and for SRS Cohen's d is reported. Rosenthal r: small effect > 0.1, moderate effect > 0.3, strong effect > 0.5. Cohen's d: small effect > 0.1, moderate effect > 0.5, strong effect > 0.8*.

* *Fisher's exact Test*.

** *Mann-Whitney U-Test. Bold values refers to statistically significant results (i.e., p < 0.05)*.

**Figure 2:**
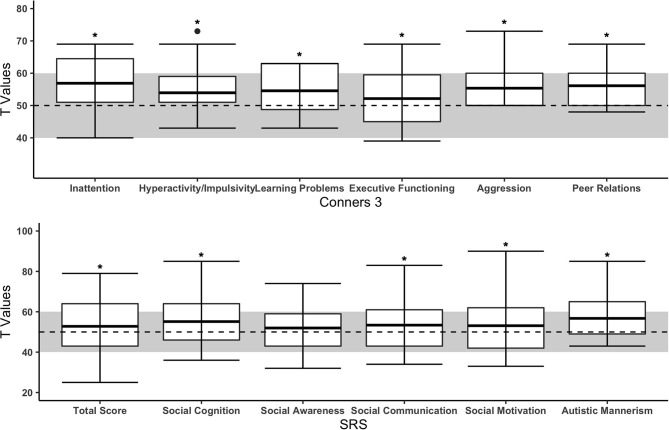
Subscales of parent-reported Conners-3 and SRS of children with CHD compared to the norm. The upper and lower borders of the box correspond to the third and first quartile. The thick line in the box represents the mean. The normal range is highlighted in gray (T = 50 ± 10). Dashed line = mean of norm (T = 50). *Significant difference between CHD and norm populations.

Boxplots for ADHD-related symptoms (Conners-3) are provided in Figure 2. All subscales of the parent-reported Conners-3 were significantly higher in children with CHD compared to the norm (see Table 2). Effect sizes ranged from small (Executive function: Rosenthal *r* = 0.24) to large (Aggression: Rosenthal *r* = 0.73). Of the 125 participating children with CHD, parents of 9 children reported that they had previously been diagnosed with ADHD. Teacher-reported Conners-3 revealed similar findings (see Supplementary Table 1).

Social interaction problems (SRS) are presented in Figure 2. Children with CHD showed significantly more problems in “social cognition,” “social communication,” “social motivation,” and “autistic mannerism” but not in the ‘social awareness’ scale (see Table 2). Effect sizes ranged from small (Total score: Cohen's *d* = 0.21) to moderate (Autistic mannerism: Cohen's *d* = 0.72). Of the 105 children with parental rating of the SRS, one child had a diagnosis of ASD.

**Figure 3:**
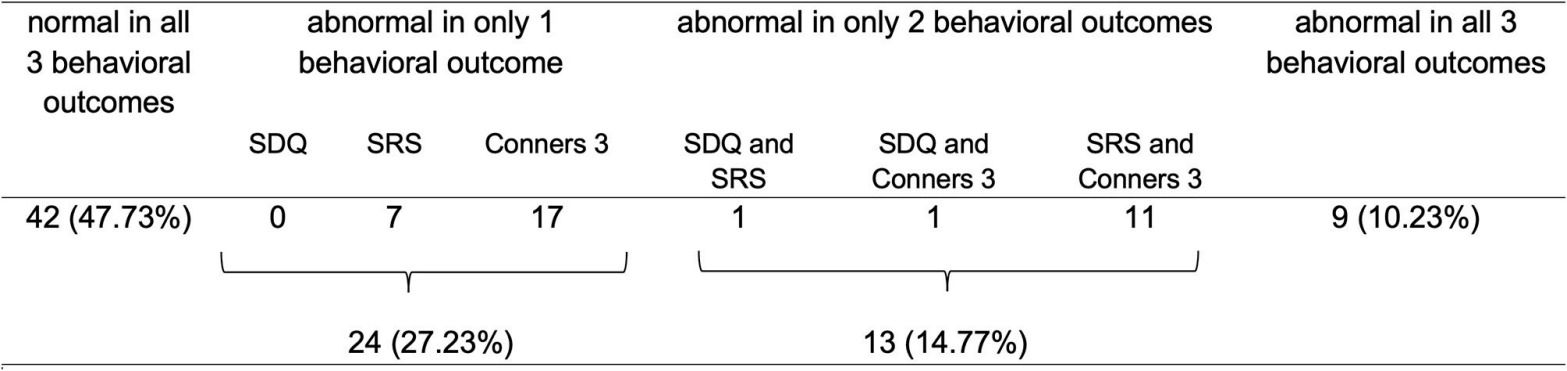
Overview of parent-reported abnormal behavior across questionnaires and comorbidities (*N* = 88). In Conners-3, 43 children had 2 or more subscales scoring outside the normal range, of which 77% included the subscales “inattention” and/or “hyperactivity/impulsivity”.

Parents of 88 children had completed all three behavioral questionnaires. Of this sub-sample, 25% (*N* = 22 of 88) showed abnormal behavior in two or three behavioral outcomes, most frequently in the Conners-3 and SRS [23% (*N* = 20 of 88); see Figure 3].

### Risk Factors Analysis

Three separate linear regression models were calculated for the association of behavioral outcomes at 10 years of age with predictive risk factors as independent variables. Statistical measures and estimates are presented in Table 3. All linear regression models indicated a moderate effect size (SDQ: adjusted *R*^2^ = 0.223, *p-*value = 0.007; SRS: adjusted *R*^2^ = 0.228, *p-*value = 0.010; Connors-3: McFadden adjusted *R*^2^ = 0.168). Lower IQ and poorer maternal mental health at 4 years of age significantly predicted more behavioral problems at 10 years of age in all linear regression models. There was no association between behavioral outcomes and any cardiac or neonatal factor, except for sex and gestational age for ADHD-related symptoms. SES was not significantly associated with behavioral outcomes in any model.

**Table 3:** Multiple linear regressions for risk factor analysis for behavioral outcome of children with CHD at 10 years of age.

**Dependent variable**	**Independent variable**	**Standardized estimate**	**CI-95**	***p*-value**
SDQ ^*^(Total Score)	Total IQ 4 years	−0.33	−0.36 to −0.31	**0.010**
	SES	−0.05	−0.19–0.09	0.692
	BSI 4 years	0.40	0.38–0.42	**0.001**
	Sex	−0.08	−0.57–0.42	0.501
	Gestational age	0.05	−0.09–0.20	0.641
	Birth weight (*z*-scores)	−0.20	−0.46–0.05	0.109
	Univentricular CHD	−0.01	−0.66–0.64	0.943
	Mean pre-operative saturation	−0.01	−0.04–0.01	0.930
	Age at first CPB surgery	−0.18	−0.27 to −0.10	0.153
	Lowest intraoperative temperature	−0.01	−0.08–0.06	0.939
	Extracorporeal circulation time	0.09	0.09–0.10	0.466
	Length of hospitalization	0.03	0.00–0.04	0.824
SRS ^*^(Total Score)	Total IQ 4 years	−0.44	−0.73 to −0.14	**0.002**
	SES	−0.09	−1.81–1.62	0.427
	BSI 4 years	0.42	0.17–0.67	**0.001**
	Sex	0.09	−6.07–6.24	0.460
	Gestational age	0.11	−1.63–1.84	0.391
	Birth weight (z-scores)	0.03	−2.94–3.00	0.829
	Univentricular CHD	−0.11	−8.07–7.86	0.415
	Mean pre-operative saturation	0.00	−0.30–0.30	0.992
	Age at first CPB surgery	0.04	−1.01–1.09	0.767
	Lowest intraoperative temperature	0.10	−0.76–0.12	0.467
	Extracorporeal circulation time	0.07	0.02–0.12	0.608
	Length of hospitalization	−0.01	−0.11–0.09	0.945
Conners-3 ^**^(Number of abnormal subscales)	Total IQ 4 years	0.93	0.89–0.97	**0.002**
	SES	0.84	0.63–1.11	0.203
	BSI 4 years	1.06	1.01–1.11	**0.013**
	Sex	4.48	1.54–13.87	**0.006**
	Gestational age	1.38	1.03–1.86	**0.030**
	Birth weight (z-scores)	1.16	0.70–1.92	0.570
	Univentricular CHD	1.14	0.30–4.14	0.846
	Mean pre-operative saturation	1.03	0.97–1.09	0.460
	Age at first CPB surgery	0.97	0.80–1.17	0.752
	Lowest intraoperative temperature	1.04	0.90–1.21	0.578
	Extracorporeal circulation time	1.00	1.00–1.01	0.343
	Length of hospitalization	1.00	0.99–1.02	0.567

* *Linear regression: standardized estimate = beta*.

** *Ordinal regression: standardized estimate = odds ratio. Bold values refers to statistically significant results (i.e., p < 0.05)*.

## Discussion

The current study investigated multidimensional behavioral outcomes in 10-year-old children with complex CHD. While the behavioral outcomes of CHD children were akin to the healthy norm population when assessed with a general screening instrument (SDQ), we found particular difficulties, such as increased ADHD-related symptoms and difficulties in social interaction, when using disorder-specific questionnaires (Conners-3, SRS). The child's intellectual abilities and the mother's mental health during early childhood were predictive for later behavioral outcomes at 10 years of age. Interestingly, neither neonatal nor cardiac-related factors were associated with later behavioral outcomes.

### Behavioral Outcome

We did not detect any behavioral difficulties when using the SDQ as a brief screening instrument for general behavioral difficulties, even if the internalizing and externalizing subscales were considered separately. In contrast, in a sample of children with CHD who were previously examined in our center at 10 years of age, the SDQ was used and more behavioral problems were reported by the parents for “emotional symptoms” and “hyperactivity/inattention” (41). This difference in reported behavioral symptoms may be due to the use of different norm groups (i.e., we used newer age and sex stratified normative data) and differences in combining behavioral subscales. Also, the cohort in Liamlahi et al. was born between 1995 and 1998 which is 10 years prior to the present study cohort and may represent a more vulnerable population due to improvements in medical care over the last decade. Further, it is also important to mention that depending on who is reporting behavioral symptoms, severity may differ. In our study, only parent and teacher reports were obtained. In a study by Schaefer et al. examining 14 year old adolescents with CHD, adolescents themselves did not report more behavioral problems in the SDQ, while parents reported more behavioral difficulties for their children (27). One study comparing CHD and preterm born adolescents reported a similar proportion of individuals with behavioral difficulties (i.e., 23.7%) (26). Concluding, a general screening tool might be too inaccurate to reliably detect subclinical behavioral difficulties in older CHD children.

Interestingly, our study demonstrates that when using disorder-specific questionnaires, ADHD-related behaviors and social interaction problems can be detected. Our finding of increased behavioral problems in CHD is in line with a recent meta-analysis by Abda et al. (4). ADHD-related symptoms measured with the Conners-3 showed significant problems in all subscales, particularly in the subscale “inattention” which is in line with the literature (42). Social interaction problems measured with the SRS were found most frequently in the subscales “social cognition” and “autistic mannerism.” “Autistic mannerism” includes autistic features such as narrow areas of interest, unusual sensory interest, and stereotypical behaviors. The “social cognition” subscale assesses the child's ability to recognize the meaning of social acts. A key element of social cognition is the development of the theory of mind. Thus, our findings are in line with previous studies reporting impairments of theory of mind in children with CHD (4). This further emphasizes the importance of assessing social abilities in this patient population in order to detect less overt difficulties, even in the absence of an ASD diagnosis.

In our cohort, the prevalence of ADHD and ASD diagnoses were similar to the general population (in our cohort: ADHD = 7%, ASD = <1%) (43, 44). However, we defined these diagnoses on the base of parent reports and we did not perform formal psychiatric testing. In fact, higher ADHD and ASD prevalences in children with CHD have been observed in other studies (5, 6, 45). Nevertheless, the discrepancy between diagnoses and subclinical pathology in CHD has been discussed before, since neurodevelopmental impairments in CHD patients are often mild to moderate and may thus remain subclinical (10, 46). However, especially the comorbidity of subclinical pathology among different domains may still lead to poorer academic achievement and psychological distress (47). Indeed, the majority of the children with CHD enrolled in our study scored within the normal range across different subscales related to ADHD-related behaviors and social interaction problems. However, scores were elevated by 0.2–0.7 standard deviations compared to the norm population and 23% of our patients showed both significant ADHD-related behaviors and difficulties in social interaction. Uekermann et al. has described the association of social cognition and ADHD and explained that inattention and hyperactivity may lead to social cognition deficits (48). Furthermore, social cognition deficits often co-occur in children with both ADHD or ASD (42).

The etiology of behavioral difficulties in CHD remains unclear. It has previously been discussed that brain alterations, caused by the cardiac defect itself, may mediate behavioral dysfunction (9). In fact, ADHD-related behavior has been associated with altered brain white matter microstructure in adolescents with CHD (49) and patients with ADHD and ASD show significant functional and structural brain alterations (50). On the other hand, behavioral difficulties may also derive from problematic parent-child interaction due to increased chronic and post-traumatic stress in parents of children with CHD (4). In turn, children themselves may experience emotional stress (5). Thus, it is of importance to better understand the underlying medical and psychosocial mechanisms of behavioral difficulties in children with CHD in order to improve the support of affected families. Accordingly, we have conducted an extensive risk factor analysis to investigate different predictors of behavioral difficulties.

### Risk Factors for Behavioral Outcome

We investigated a range of psychological, social, neonatal, and cardiac-related risk factors for adverse behavioral outcomes. Child's intellectual abilities (i.e., IQ) and mother's mental health during early childhood were consistently predictive for all behavioral outcomes at 10 years of age. Furthermore, sex and gestational age were predictive for ADHD-related symptoms. Interestingly, other neonatal and medical factors (i.e., peri- and post-operative conditions and CHD severity), as well as SES were not associated with any behavioral outcomes.

The association between IQ and behavior in children with CHD has been shown previously (14, 51). Furthermore, the association between IQ, sex, and gestational age with ADHD-related symptoms in children with CHD is in line with the literature (52, 53). In addition, the male sex is also associated with a higher prevalence for ADHD in the norm population (54). The impact of parental mental health on long-term neurodevelopment in children with CHD has been discussed in a recent review by Ryan et al. (55). Indeed, an article by McCusker elaborated that maternal mental health and other family-related factors excel the impact of medical risk factors on the behavioral outcome in children with CHD (56). The current study demonstrates that parental mental health during early childhood has a bearing on different aspects of behavior, including social interaction and ADHD-related symptoms, even years later. However, this might be not specific for children with CHD as the influence of parental mental health on child's behavior has been shown in the norm population as well (57). These findings stress the importance of identifying families at risk for mental health problems at an early stage and supporting them accordingly.

Surprisingly, SES was not associated with any behavioral outcome, even when IQ and maternal mental health, which might share explained variance with SES (58, 59), were excluded from the model. This finding is inconsistent with a review by Cassidy et al. (5). Reasons for this difference could be the overall high SES in our cohort and the resulting small variance between families. Furthermore, SES did not include measures like income and neighborhood which is often assessed in cohorts from North America, where SES has been described as one of the strongest predictors for impaired behavior in children with CHD (5).

Interestingly, perioperative and cardiac risk factors were not predictive for later behavioral outcomes. This is in accordance with a recent meta-analysis by Huisenga et al., which demonstrated that findings on perioperative risk factors predicting developmental outcomes are inconsistent and that other factors may outweigh the contribution of medical risk factors (6). Reasons why we did not find any association between medical risk factors and behavior, although it was inconsistent in the meta-analysis, may be that our cohort differs from studies of the meta-analysis regarding inclusion criteria and methodological approach. The meta-analysis only included studies with children having the first cardiac surgery in the first 9 weeks of life, whereas we included children with the first cardiac surgery up to 6 years of age. In addition, we explicitly ruled out collinearity between risk factors, which was quite common and might have been neglected in previous studies.

### Strengths and Limitations

There is already a considerable number of studies looking at behavioral outcomes of children with CHD. However, to our knowledge, this is the first study on a broad spectrum of CHD patients looking at different behavioral outcomes, including ADHD-related symptoms and social interaction problems and, furthermore, testing various risk factors including both, medical and psychosocial risk factors as predictors for multidimensional behavioral outcomes. Nevertheless, this study has some limitations. The study was conducted at a single center, which limits the generalizability of the results. However, as this center is specialized in cardiac surgery, children from a large geographical area of Switzerland are being operated at this center and are thus included in this study sample. The questionnaires were missing in some families (SDQ *N* = 123, Conners-3 *N* = 98, SRS *N* = 105) and were only compared to test norms because of a missing control group. Consequently, the rates of comorbidities could not be compared to a normal population. Furthermore, there were no formal diagnostic assessments, in particular of social function and ADHD, performed. In addition, no self-reports were obtained and mental well-being of the parents might negatively influence their ratings. However, we had questionnaires completed by parents and teachers, and the extent of rated behavioral problems was comparable. As mentioned before, we investigated a high functioning sample with relatively high SES and IQ compared to the patients lost to follow-up. Further, the sample lacks a heterogeneity of ethnicities. Since we still found significant behavioral problems in this sample, it can be assumed that behavioral problems might be even more pronounced in the general CHD sample. Another limitation is the lack of a specific questionnaire for internalizing behavior investigating anxiety and depressive symptoms in CHD children, as well as evaluating perception of illness in parents and children as this may add additional information on the burden of the disease. Furthermore, while we included different types of CHD with varying degrees of complexity, the relatively small sample size did not allow to examine behavior within specific heart diseases.

## Conclusion

This study provides further evidence that school-aged children with CHD are at increased risk for ADHD-related symptoms and difficulties in social interaction. Importantly, the use of disorder-specific questionnaires is strongly recommended for comprehensive assessments of subclinical and clinical difficulties. Particular focus should be placed on social cognition as difficulties in this domain may not become clinically recognizable. Behavioral problems are most prominent in children with low IQ and mothers with mental health issues, which has been previously described in other populations. Therefore, we emphasize the importance to carefully screen families, especially mothers, for mental health problems, as early interventions may improve behavioral outcome of CHD patients.

## Data Availability Statement

The raw data supporting the conclusions of this article will be made available by the authors, without undue reservation.

## Ethics Statement

The studies involving human participants were reviewed and approved by Ethical committee of canton Zurich KEK-ZH-Nr. 2014-0071. Written informed consent to participate in this study was provided by the participants' legal guardian/next of kin.

## Author Contributions

IW analyzed and interpreted the data, and drafted the initial manuscript. ME contributed in data analysis, interpretation, and editing the manuscript. FW interpreted the data and critically revised the manuscript. SP assessed the data and critically revised the manuscript. ML designed the questionnaires and critically revised the manuscript. EV critically revised the manuscript. BL designed the study, contributed in data interpretation, and critically revised the manuscript. All authors read and approved the final manuscript for submission.

## Conflict of Interest

The authors declare that the research was conducted in the absence of any commercial or financial relationships that could be construed as a potential conflict of interest.

## Abbreviations

ADHD: Attention deficit hyperactivity disorder
ASD: Autism Spectrum Disorder
BSI: Brief Symptom Inventory
CHD: congenital heart disease
CPB: cardiopulmonary bypass
GA: gestational age
ICU: intensive care unit
SDQ: Strength and Difficulties Questionnaire
SES: socioeconomic status
SRS: Social Responsiveness Scale.

## References

[1] Bernier PL Stefanescu A Samoukovic G Tchervenkov CI. The challenge of congenital heart disease worldwide: epidemiologic and demographic facts. Semin Thorac Cardiovasc Surg Pediatr Card Surg Annu. (2010) 13:26–34. 10.1053/j.pcsu.2010.02.005 20307858

[2] Hoffman JIE Kaplan S. The incidence of congenital heart disease. J Am Coll Cardiol. (2002) 39:1890–900. 10.1016/S0735-1097(02)01886-7 12084585

[3] Khairy P Ionescu-Ittu R Mackie AS Abrahamowicz M Pilote L Marelli AJ. Changing mortality in congenital heart disease. J Am Coll Cardiol. (2010) 56:1149–57. 10.1016/j.jacc.2010.03.085 20863956

[4] Abda A Bolduc ME Tsimicalis A Rennick J Vatcher D Brossard-Racine M. Psychosocial outcomes of children and adolescents with severe congenital heart defect: a systematic review and meta-analysis. J Pediatr Psychol. (2019) 44:463–77. 10.1093/jpepsy/jsy085 30452652

[5] Cassidy AR Ilardi D Bowen SR Hampton LE Heinrich KP Loman MM. Congenital heart disease: a primer for the pediatric neuropsychologist. Child Neuropsychol. (2018) 24:859–902. 10.1080/09297049.2017.1373758 28874075

[6] Huisenga D La Bastide-Van Gemert S Van Bergen A Sweeney J Hadders-Alegra M. Developmental outcomes after early surgery for complex congenital heart disease: a systematic review and meta-analysis. Dev Med Child Neurol. (2020). 10.1111/dmcn.14512. [Epub ahead of print]. 32149404 PMC7754445

[7] Karsdorp PA Everaerd W Kindt M Mulder BJ. Psychological and cognitive functioning in children and adolescents with congenital heart disease: a meta-analysis. J Pediatr Psychol. (2007) 32:527–41. 10.1093/jpepsy/jsl047 17182669

[8] Clancy T Jordan B de Weerth C Muscara F. Early emotional, behavioural and social development of infants and young children with congenital heart disease: a systematic review. J Clin Psychol Med Sett. (2019) 27:686–703. 10.1007/s10880-019-09651-1 31506852

[9] Bellinger DC. Are children with congenital cardiac malformations at increased risk of deficits in social cognition? Cardiol Young. (2008) 18:3–9. 10.1017/S104795110700176X 18093362

[10] Bean Jaworski JL Flynn T Burnham N Chittams JL Sammarco T Gerdes M. Rates of autism and potential risk factors in children with congenital heart defects. Congenit Heart Dis. (2017) 12:421–9. 10.1111/chd.12461 28299880 PMC5548597

[11] Hövels-Gürich HH. Factors influencing neurodevelopment after cardiac surgery during infancy. Front Pediatr. (2016) 4:137. 10.3389/fped.2016.00137 28018896 PMC5156661

[12] Hövels-Gürich HH Konrad K Wiesner M Minkenberg R Herpertz-Dahlmann B Messmer BJ. Long term behavioural outcome after neonatal arterial switch operation for transposition of the great arteries. Arch Dis Child. (2002) 87:506–10. 10.1136/adc.87.6.506 12456550 PMC1755850

[13] Gaynor JW Stopp C Wypij D Andropoulos DB Atallah J Atz AM Impact of operative and postoperative factors on neurodevelopmental outcomes after cardiac operations. Ann Thorac Surg. (2016) 102:843–9. 10.1016/j.athoracsur.2016.05.081 27496628

[14] Holland JE Cassidy AR Stopp C White MT Bellinger DC Rivkin MJ Psychiatric disorders and function in adolescents with tetralogy of fallot. J Pediatr. (2017) 187:165–73. 10.1016/j.jpeds.2017.04.048 28533034

[15] Spijkerboer AW De Koning WB Duivenvoorden HJ Bogers AJ Verhulst FC Helbing WA. Medical predictors for long-term behavioral and emotional outcomes in children and adolescents after invasive treatment of congenital heart disease. J Pediatr Surg. (2010) 45:2146–53. 10.1016/j.jpedsurg.2010.07.026 21034936

[16] McCusker CG Armstrong MP Mullen M Doherty NN Casey FA. A sibling-controlled, prospective study of outcomes at home and school in children with severe congenital heart disease. Cardiol Young. (2013) 23:507–16. 10.1017/S1047951112001667 23083543

[17] Woolf-King SE Anger A Arnold EA Weiss SJ Teitel D. Mental health among parents of children with critical congenital heart defects: a systematic review. J Am Heart Assoc. (2017) 6:e004862. 10.1161/JAHA.116.004862 28151402 PMC5523775

[18] Landolt MA Ystrom E Stene-Larsen K Holmstrom H Vollrath ME. Exploring causal pathways of child behavior and maternal mental health in families with a child with congenital heart disease: a longitudinal study. Psychol Med. (2014) 44:3421–33. 10.1017/S0033291713002894 24286537

[19] Naef N Wehrle F Rousson V Latal B. Cohort and individual neurodevelopmental stability between 1 and 6 years of age in children with congenital heart disease. J Pediatr. (2019) 215:83–9. 10.1016/j.jpeds.2019.08.036 31563274

[20] Jenkins KJ Gauvreau K Newburger JW Spray TL Moller JH Iezzoni LI. Consensus-based method for risk adjustment for surgery for congenital heart disease. J Thorac Cardiovasc Surg. (2002) 123:110–8. 10.1067/mtc.2002.119064 11782764

[21] Goodman R. Psychometric properties of the strengths and difficulties questionnaire. J Am Acad Child Adolesc Psychiatry. (2001) 40:1337–45. 10.1097/00004583-200111000-00015 11699809

[22] Lohbeck A Schultheiß J Petermann F Petermann U Die Deutsche selbstbeurteilungsversion des strengths and difficulties questionnaire (SDQ-Deu-S). Diagnostica. (2015) 61:222–35. 10.1026/0012-1924/a000153

[23] Goodman A Lamping DL Ploubidis GB. When to use broader internalising and externalising subscales instead of the hypothesised five subscales on the Strengths and Difficulties Questionnaire (SDQ): data from British parents, teachers and children. J Abnorm Child Psychol. (2010) 38:1179–91. 10.1007/s10802-010-9434-x 20623175

[24] Bölte S. Brief Report: the Social Responsiveness Scale for Adults (SRS-A): initial results in a German cohort. J Autism Dev Disord. (2012) 42:1998–9. 10.1007/s10803-011-1424-5 22183423 PMC3425739

[25] Lidzba K Christiansen H Drechsler R Conners-3. Conners Skalen zu Aufmerksamkeit und Verhalten – 3. Deutschsprachige Adaptation der Conners 3rd EditionTM (Conners 3TM) von C. Keith Conners. Lidzba, Karen; Christiansen, Hanna; Drechsler, Renate (2013) Conners-3 Conners Skalen zu Aufmerksamkeit und Verhalten – 3 Deutschsprachige Adaptation der Conners 3rd EditionTM (Conners 3TM) von C Keith Conners. Bern: Verlag Hans Huber (2013).

[26] Easson K Dahan-Oliel N Rohlicek C Sahakian S Brossard-Racine M Mazer B A comparison of developmental outcomes of adolescent neonatal intensive care unit survivors born with a congenital heart defect or born preterm. J Pediatrics. (2019) 207:34–41. 10.1016/j.jpeds.2018.11.002 30528759

[27] Schaefer C von Rhein M Knirsch W Huber R Natalucci G Caflisch J. Neurodevelopmental outcome, psychological adjustment, and quality of life in adolescents with congenital heart disease. Dev Med Child Neurol. (2013) 55:1143–9. 10.1111/dmcn.12242 23937239

[28] Calderon J Bellinger DC Hartigan C Lord A Stopp C Wypij D. Improving neurodevelopmental outcomes in children with congenital heart disease: protocol for a randomised controlled trial of working memory training. BMJ Open. (2019) 9:e023304-e. 10.1136/bmjopen-2018-023304 30782877 PMC6377570

[29] Taurines R Schwenck C Westerwald E Sachse M Siniatchkin M Freitag C ADHD and autism: differential diagnosis or overlapping traits? A selective review. Atten Defic Hyperact Disord. (2012) 4:115–39. 10.1007/s12402-012-0086-2 22851255

[30] Wotherspoon JM Eagleson KJ Gilmore L Auld B Hirst A Johnson S. Neurodevelopmental and health-related quality-of-life outcomes in adolescence after surgery for congenital heart disease in infancy. Dev Med Child Neurol. (2020) 62:214–20. 10.1111/dmcn.14251 31025336

[31] Franke GH Brief Symptom Inventory (BSI) von LR Derogatis:(Kurzform der SCL-90-R). Göttingen: Beltz Test (2000).

[32] Ricken GF Schuck A Preuss KD Hannover-Wechsler-Intelligenztest für das Vorschulalter. Bern: Huber (2007).

[33] Largo RH Pfister D Molinari L Kundu S Lipp A Duc G. Significance of prenatal, perinatal and postnatal factors in the development of AGA preterm infants at five to seven years. Dev Med Child Neurol. (1989) 31:440–56. 10.1111/j.1469-8749.1989.tb04022.x 2680687

[34] Janitza S Klipker K Hölling H. Age-specific norms and validation of the German SDQ parent version based on a nationally representative sample (KiGGS). Eur Child Adolesc Psychiatry. (2019) 29:123–36. 10.1007/s00787-019-01337-1 31016398

[35] Bölte S SRS: Skala zur Erfassung sozialer Reaktivität : dimensionale Autismus-Diagnostik. In: Poustka F, editor. Deutsche Fassung der Social Responsiveness Scale (SRS) / von John N. Constantino und Christian P. Gruber. Bern: Huber (2008).

[36] Goodman R. The strengths and difficulties questionnaire: a research note. J Child Psychol Psychiatry. (1997) 38:581–6. 10.1111/j.1469-7610.1997.tb01545.x 9255702

[37] Calderon J Willaime M Lelong N Bonnet D Houyel L Ballon M. Population-based study of cognitive outcomes in congenital heart defects. Arch Dis Child. (2018) 103:49–56. 10.1136/archdischild-2016-310830 28780508

[38] Marino Bradley S Lipkin Paul H Newburger Jane W Peacock G Gerdes M Gaynor JW. Neurodevelopmental outcomes in children with congenital heart disease: evaluation and management. Circulation. (2012) 126:1143–72. 10.1161/CIR.0b013e318265ee8a 22851541

[39] Vatcheva KP Lee M McCormick JB Rahbar MH. Multicollinearity in regression analyses conducted in epidemiologic studies. Epidemiology. (2016) 6:227. 10.4172/2161-1165.1000227 27274911 PMC4888898

[40] R Core Team R: A Language and Environment for Statistical Computing. Vienna: R Foundation for Statistical Computing (2020).

[41] Liamlahi R von Rhein M Buhrer S Valsangiacomo Buechel ER Knirsch W Landolt MA. Motor dysfunction and behavioural problems frequently coexist with congenital heart disease in school-age children. Acta Paediatr. (2014) 103:752–8. 10.1111/apa.12639 24661017

[42] Miranda A Berenguer C Roselló B Baixauli I Colomer C. Social cognition in children with high-functioning autism spectrum disorder and attention-deficit/hyperactivity disorder. associations with executive functions. Front Psychol. (2017) 8:1035. 10.3389/fpsyg.2017.01035 28690570 PMC5481358

[43] Elsabbagh M Divan G Koh Y-J Kim YS Kauchali S Marcín C. Global prevalence of autism and other pervasive developmental disorders. Autism Res. (2012) 5:160–79. 10.1002/aur.239 22495912 PMC3763210

[44] Polanczyk GV Willcutt EG Salum GA Kieling C Rohde LA. ADHD prevalence estimates across three decades: an updated systematic review and meta-regression analysis. Int J Epidemiol. (2014) 43:434–42. 10.1093/ije/dyt261 24464188 PMC4817588

[45] Tsao PC Lee YS Jeng MJ Hsu JW Huang KL Tsai SJ. Additive effect of congenital heart disease and early developmental disorders on attention-deficit/hyperactivity disorder and autism spectrum disorder: a nationwide population-based longitudinal study. Eur Child Adolesc Psychiatry. (2017) 26:1351–9. 10.1007/s00787-017-0989-8 28417257

[46] Calderon J Bellinger DC. Executive function deficits in congenital heart disease: why is intervention important? Cardiology Young. (2015) 25:1238–46. 10.1017/S1047951115001134 26082199

[47] Latal B. Neurodevelopmental outcomes of the child with congenital heart disease. Clin Perinatol. (2016) 43:173–85. 10.1016/j.clp.2015.11.012 26876129

[48] Uekermann J Kraemer M Abdel-Hamid M Schimmelmann BG Hebebrand J Daum I. Social cognition in attention-deficit hyperactivity disorder (ADHD). Neurosci Biobehav Rev. (2010) 34:734–43. 10.1016/j.neubiorev.2009.10.009 19857516

[49] Rollins CK Watson CG Asaro LA Wypij D Vajapeyam S Bellinger DC. White matter microstructure and cognition in adolescents with congenital heart disease. J Pediatr. (2014) 165:936–44. 10.1016/j.jpeds.2014.07.028 25217200 PMC4258111

[50] Lukito S Norman L Carlisi C Radua J Hart H Simonoff E. Comparative meta-analyses of brain structural and functional abnormalities during cognitive control in attention-deficit/hyperactivity disorder and autism spectrum disorder. Psychol Med. (2020) 50:894–919. 10.1017/S0033291720000574 32216846 PMC7212063

[51] Bellinger DC Newburger JW Wypij D Kuban KC duPlesssis AJ Rappaport LA. Behaviour at eight years in children with surgically corrected transposition: the Boston Circulatory Arrest Trial. Cardiol Young. (2009) 19:86–97. 10.1017/S1047951108003454 19079812 PMC4942187

[52] DeMaso DR Calderon J Taylor GA Holland JE Stopp C White MT. Psychiatric disorders in adolescents with single ventricle congenital heart disease. Pediatrics. (2017) 139:e20162241. 10.1542/peds.2016-2241 28148729 PMC5330395

[53] Calderon J Stopp C Wypij D DeMaso DR Rivkin M Newburger JW. Early-term birth in single-ventricle congenital heart disease after the fontan procedure: neurodevelopmental and psychiatric outcomes. J Pediatr. (2016) 179:96–103. 10.1016/j.jpeds.2016.08.084 27692462

[54] Thapar A Cooper M. Attention deficit hyperactivity disorder. Lancet. (2016) 387:1240–50. 10.1016/S0140-6736(15)00238-X 26386541

[55] Ryan KR Jones JB Allen KY Marino BS Casey F Wernovsky G. Neurodevelopmental outcomes among children with congenital heart disease: at-risk populations and modifiable risk factors. World J Pediatr Congenit Heart Surg. (2019) 10:750–8. 10.1177/2150135119878702 31658880

[56] McCusker CG Doherty NN Molloy B Casey F Rooney N Mulholland C. Determinants of neuropsychological and behavioural outcomes in early childhood survivors of congenital heart disease. Arch Dis Child. (2007) 92:137–41. 10.1136/adc.2005.092320 17030557 PMC2083334

[57] Prady SL Pickett KE Croudace T Mason D Petherick ES McEachan RRC. Maternal psychological distress in primary care and association with child behavioural outcomes at age three. Eur Child Adolesc Psychiatry. (2016) 25:601–13. 10.1007/s00787-015-0777-2 26415597 PMC4889639

[58] Oommen A Factors influencing intelligence quotient. J Neurol Stroke. (2014) 1:1–5. 10.15406/jnsk.2014.01.00023

[59] Hardie JH Landale NS. Profiles of risk: maternal health, socioeconomic status, and child health. J Marriage Fam. (2013) 75:651–66. 10.1111/jomf.12021 23794751 PMC3685849

