# Supplementary material for: Social and Behavioral Difficulties in 10-Year-Old Children With Congenital Heart Disease: Prevalence and Risk Factors
Source: Front Pediatr. 2020 Dec 11;8:604918. doi: 10.3389/fped.2020.604918 (PMC7759662; doi:10.3389/fped.2020.604918)
Supplement: Supplementary file 1 [file Data_Sheet_1.docx]

Supplementary Material

# Supplementary Figures and Tables

## Supplementary Figures


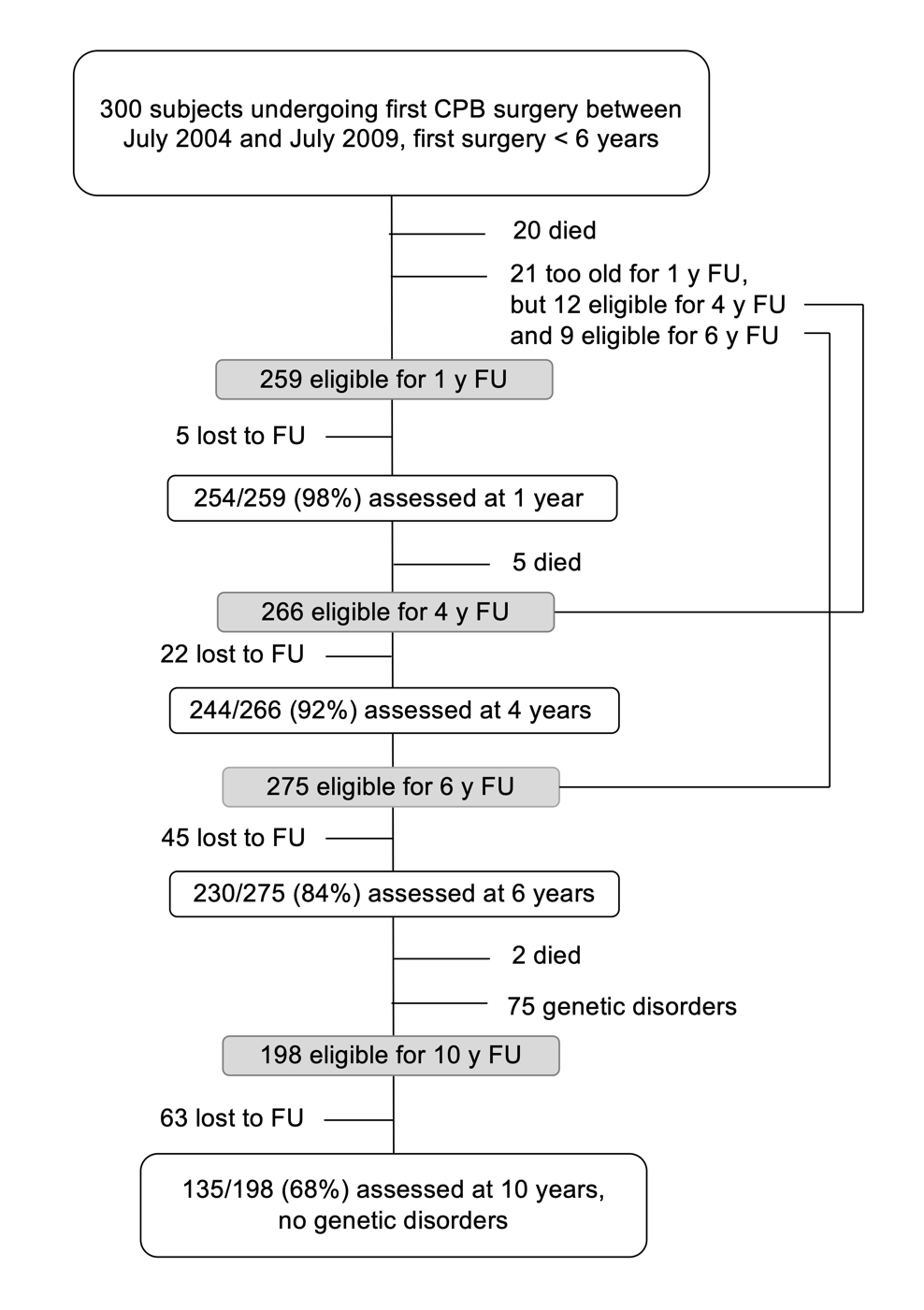


**Supplementary Figure 1.** Flow sheet.

**
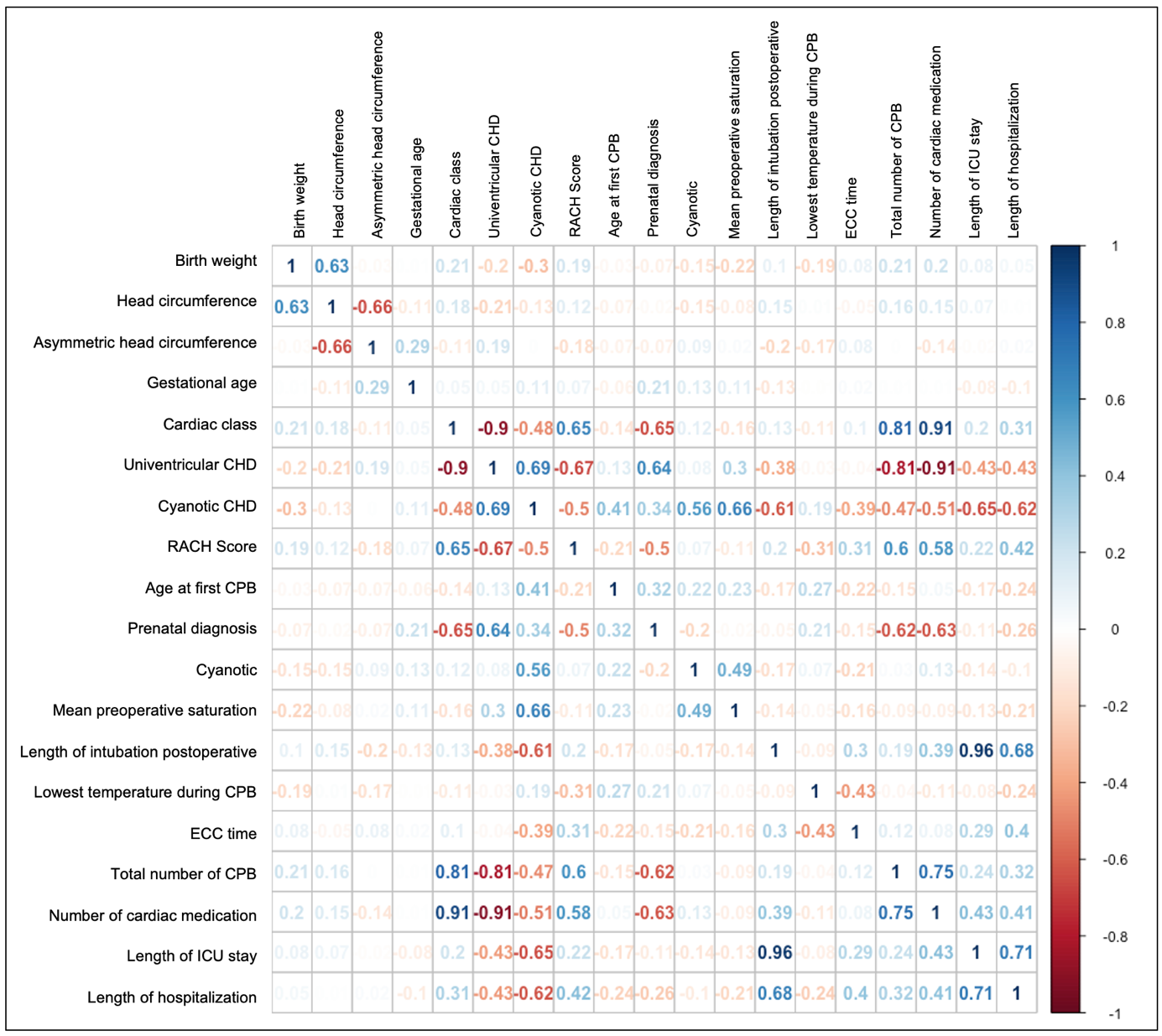
**

**Supplementary Figure 2.** Correlation matrix with all variables of interests.

## Supplementary Tables

**Supplementary Table 1.** Teacher-reported behavioral outcome of children with CHD at 10 years of age.

|  | % with abnormal Score | Mean T-Scores | Standard Estimate | *CI*- 95 | *p*- values | Effect size |
| --- | --- | --- | --- | --- | --- | --- |
| SDQ * (N=119) |  |  |  |  |  |  |
| Total Score | 24.1 |  | 0.79 | 0.51 to 1.25 | 0.292 | - |
| Internalizing Score | 21.6 |  | 0.77 | 0.49 to 1.25 | 0.268 | - |
| Externalizing Score | 24.1 |  | 0.80 | 0.51 to 1.27 | 0.297 | - |
|  |  |  |  |  |  |  |
| Conners-3 ** (N=85) |  |  |  |  |  |  |
| Inattention | 38.6 | 57.00 | 2716 | 54.50 to 59.00 | **< 0.001** | 0.58 |
| Hyperactivity/impulsivity | 9.6 | 56.00 | 2242 | 53.50 to 58.00 | **< 0.001** | 0.62 |
| Learning problems/ Executive functioning | 13.3 | 56.50 | 3242 | 55.50 to 57.00 | **< 0.001** | 0.76 |
| Aggression | 14.5 | 61.48 | 666 | 59.50 to 63.00 | **< 0.001** | 0.65 |
| Peer relations | 3.6 | 53.50 | 1715 | 52.50 to 55.50 | **< 0.001** | 0.44 |
|  |  |  |  |  |  |  |

*Note.* Scores of the SDQ are divided into normal (close to average) and abnormal (slightly raised, high, very high). Standard estimates are *odds ratio* for SDQ and *V*- values for Conners-3. Effect sizes are reported for all significant *p* values. For Conners-3 the Rosenthal *r* is reported. Rosenthal r: small effect > 0.1, moderate effect > 0.3, strong effect > 0.5. * Fisher’s exact Test. ** Mann-Whitney U Test.
